# Supplementary material for: The Effects of Peripubertal THC Exposure in Neurodevelopmental Rat Models of Psychopathology
Source: Int J Mol Sci. 2023 Feb 15;24(4):3907. doi: 10.3390/ijms24043907 (PMC9962163; doi:10.3390/ijms24043907)
Supplement: Supplementary file 1 [file ijms-24-03907-s001.zip › ijms-2134496-supplementary.pdf]

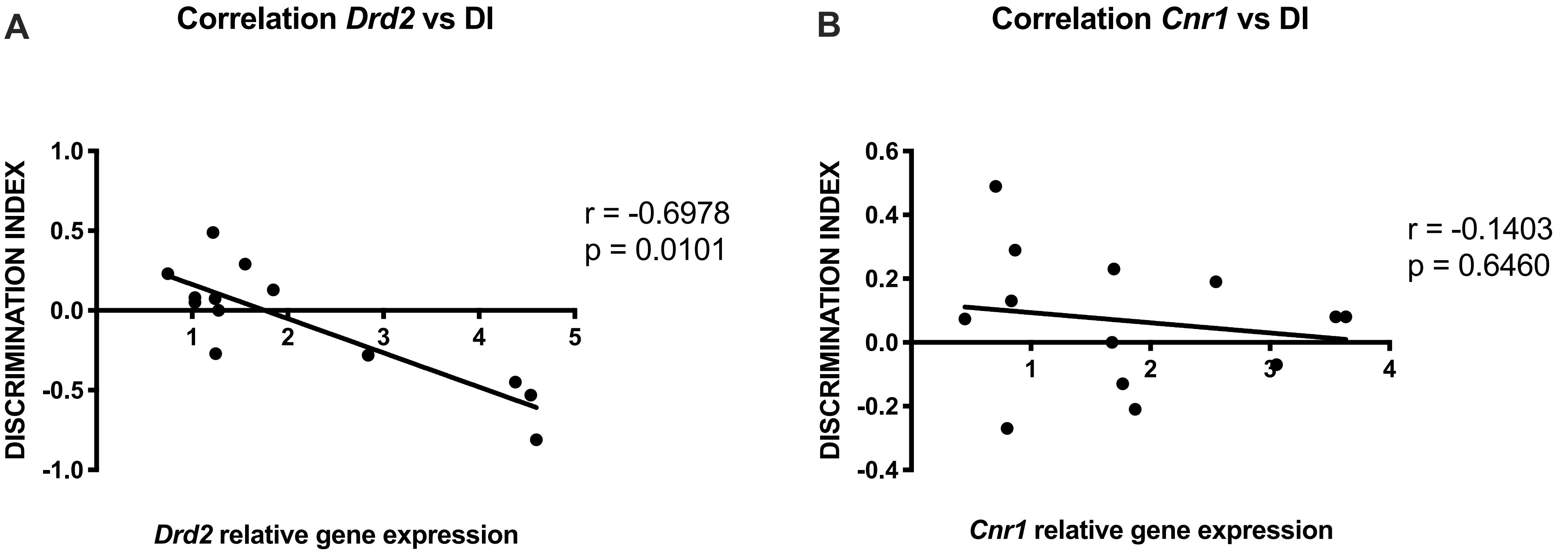
A
Correlation Drd2 vs DI
DISCRIMINATION INDEX
1.0
0.5
0.0
-0.5
-1.0
1
2
3
4
5
r = -0.6978
p = 0.0101
Drd2 relative gene expression
B
Correlation Cnr1 vs DI
DISCRIMINATION INDEX
0.6
0.4
0.2
0.0
-0.2
-0.4
1
2
3
4
r = -0.1403
p = 0.6460
Cnr1 relative gene expression

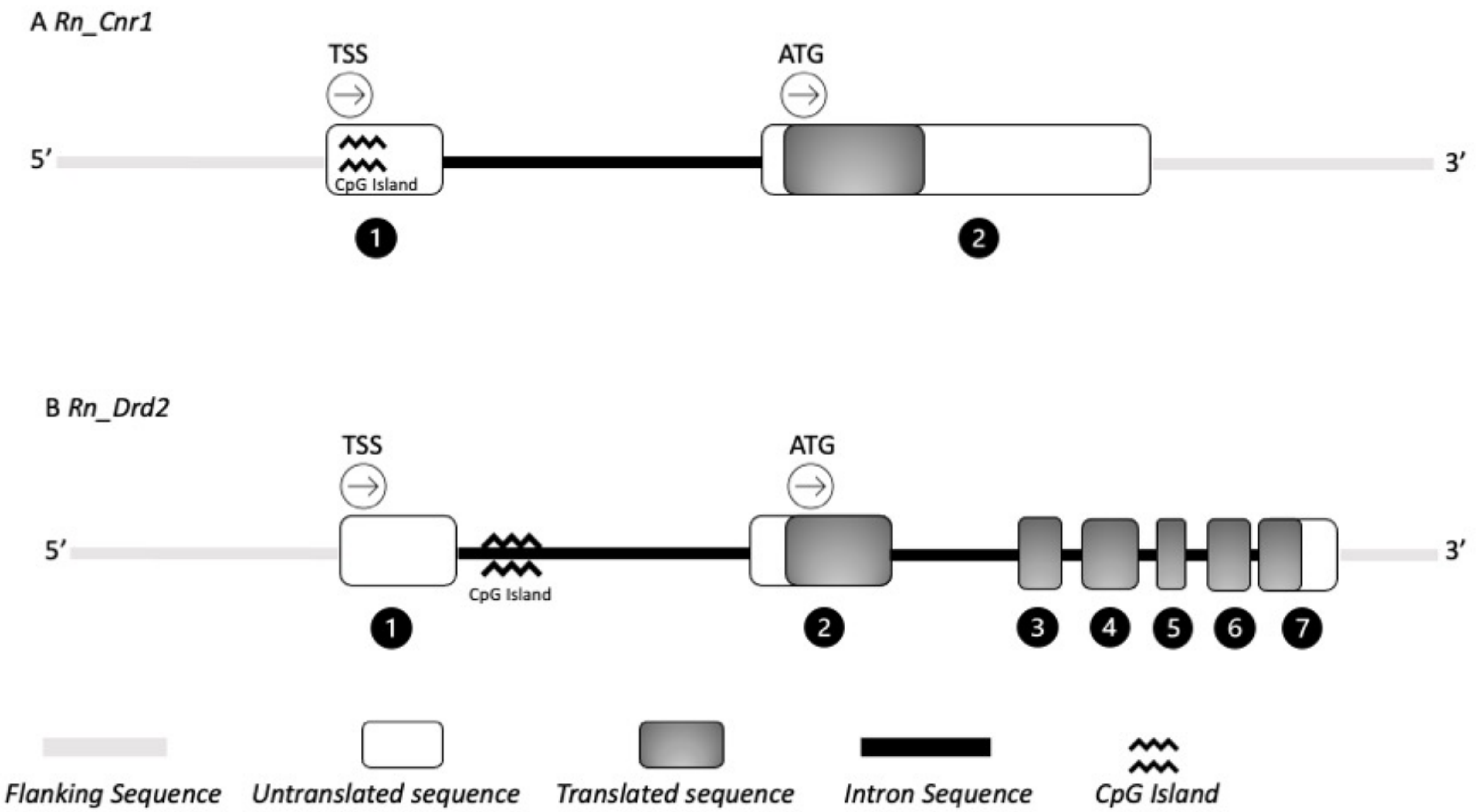
A Rn_Cnr1
TSS
ATG
5′
3′
CpG Island
1
2
B Rn_Drd2
TSS
ATG
5′
3′
CpG Island
1
2
3
4
5
6
7
Flanking Sequence
Untranslated sequence
Translated sequence
Intron Sequence
CpG Island

SUPPLEMENTARY FIGURE LEGEND

Figure S1: Correlation between *Drd2* (**A)** and *Cnr1* (**B**) gene expression in the prefrontal cortex (PFC) of MAM rats and the discrimination index (DI) in the Novel Object Recognition (NOR) test.

Figure S2: Schematic representation of rat *Cnr1*(A) and *Drd2* (B) genes. Position of transcription start site (TSS), translation start code (ATG), exons and introns, CpG islands are depicted. Details of the sequences under study for DNA methylation are shown in supplementary table S2.

Supplementary Table S1

Gene		*5'-Forward Primer-3'*	*5'-Reverse Primer-3'*
**Housekeeping**	*ß-Actin*	AGATCAAGATCATTGCTCCTCCT	ACGCAGCTCAGTAACAGTCC
	Gapdh	AGACAGCCGCATCTTCTTGT	CTTGCCGTGGGTAGAGTCAT
**Endocannabinoid System**	*Cnr1*	TTCCACCGTAAAGACAGCCC	TCCACATCAGGCAAAAGGCC
**Dopaminergic System**	*Drd2*	TACGTGCCCTTCATCGTCAC	GTGGGTACAGTTGCCCTTGA
	Drd3	GGGGTGACTGTCCTGGTCTA	AAGCCAGGTCTGATACTGAT

List of primers used for quantitative real-time RT–PCR.

Supplementary Table S2

Gene	*Sequence Analyzed*	*CpG Sites*	*5'-Primers-3'*
Rn_Cnr1	tagcttttc**cg**cctccc**cg**cccccct**cg**atactggccagtggtccccaggtgtccta**cg**	4	F: AGAAGGGTAAGATTTGGTATAGTG
			Biot_R: AACTATACAACTAAATAAACACCACATTA
			S: GTGGAGTTTGGGAATAGTTT
Rn_Drd2	ttccc**cg**a**cg**ccc**cg**agg**cg**caatctgccc**cg**t**cg**ga	6	Qiagen Assay PM00586096

Details of sequences and primers employed for the DNA methylation analysis by pyrosequencing. Rn=Rat; CpG=C-phosphate-G; F=forward primer; Biot_R= biotinylated reverse primer; S=sequencing primer; Bold text=CpG sites analyzed.
